# Knockdown lncRNA CRNDE enhances temozolomide chemosensitivity by regulating autophagy in glioblastoma

**DOI:** 10.1186/s12935-021-02153-x

**Published:** 2021-08-28

**Authors:** Zijin Zhao, Miaomiao Liu, Wenyong Long, Jian Yuan, Haoyu Li, Chi Zhang, Guodong Tang, Weixi Jiang, Xianrui Yuan, Minghua Wu, Qing Liu

**Affiliations:** 1 Department of Neurosurgery, Xiangya Hospital, Central South University, 87 Xiangya Road, Changsha, 410008 Hunan China; 2 Neurosurgical Medical Central, Central South University, Changsha, China; 3 Clinical Research Center For Skull Base Surgery and Neuro-Oncology In Hunan Province, Changsha, China; 4 Department of Nuclear Medicine (PET-CT Central), Xiangya Hospital, Central South University, Changsha, China; 5 The Key Laboratory of Carcinogenesis of the Chinese Ministry of Health, The Key Laboratory of Carcinogenesis and Cancer Invasion of the Chinese Ministry of Education, Cancer Research Institute, Central South University, Changsha, China

**Keywords:** CRNDE, Glioblastoma multiforme, Temozolomide, Chemosensitivity, Autophagy, PI3K/Akt/mTOR

## Abstract

**Background:**

The regulatory roles of long non-coding RNA (lncRNA) CRNDE in temozolomide (TMZ) chemoresistance to glioblastoma multiforme (GBM) are still poorly understood. Therefore, the function, characteristics, and possible mechanism of CRNDE in TMZ-induced chemoresistance to GBM were explored.

**Methods:**

Firstly, the expression level of CRNDE in 58 cases of glioma tissue specimens and 30 cases of normal brain tissues were tested by qRT-PCR. Meanwhile, the correlation between CRNDE expression level, the clinicopathological characteristics, and survival time of patients with glioma were analyzed. Then, the CRNDE expression in various glioma cell lines was detected, and CRNDE knockdown cell models were constructed. Subsequently, to explore the effect of CRNDE on chemosensitivity to TMZ, cell viability was detected by the CCK-8 assay and IC_50_ values, and cell proliferation was detected by cell clone assay and EdU assay, as well as cell survival was detected by apoptosis with flow cytometry under TMZ treatment. Further, the expression of drug-resistance protein ABCG2, autophagy related proteins, and PI3K/Akt/mTOR pathway were measured by western blot or qRT-PCR in TMZ-treated glioma cells. Finally, the mouse tumor xenograft model was established and the tumor volume and weight were measured, and ABCG2 expression was conducted by immunohistochemistry assay.

**Results:**

The integrated results demonstrated lncRNA CRNDE was a poor prognosis factor for GBM patient, which was upregulated in patients who were resistant to TMZ, and closely associated with chemotherapeutic response status to TMZ treatment. Further, functional assays revealed that knockdown of CRNDE could notably reduce glioma cell viability and proliferation, and elevate cell apoptosis to enhance the chemosensitivity to TMZ in vitro and in vivo. Mechanistically, the depression of CRNDE could diminish the expression of LC3 II/I, Beclin1 and Atg5 and increase the p62 expression level to inhibit autophagy due to the activation of PI3K/Akt/mTOR pathway as well as highly correlated with ABCG2 expression.

**Conclusions:**

Overall, the study provided that lncRNA CRNDE is a reliable clinical predictor of outcome and prognosis and a potential biomarker for predicting TMZ treatment response in GBM by modulating the autophagy through PI3K/Akt/mTOR pathway and ABCG2 expression which may be a novel therapeutic target for regulating TMZ sensitivity to GBM.

**Supplementary Information:**

The online version contains supplementary material available at 10.1186/s12935-021-02153-x.

## Background

Glioma is one of the most common primary brain tumors, and glioblastoma multiforme (GBM) accounting for approximately 70% of gliomas in adults is the most malignant and aggressive brain tumor, resulted in poor clinical outcomes and prognosis due to the highly invasive growth pattern and the frequent resistance to therapies [1, 2]. Despite the development of multimodal and progressive treatments including aggressive surgical resection, local radiotherapy, systemic chemotherapy, various immunotherapy, and other comprehensive methods in the past decades, the improvement of therapeutic effect is still limited with a median survival of 14.6 months and less than 3% of the 5-year survival rate in patients [1, 3]. The temozolomide-based (TMZ-based) chemotherapy is regarded as the first-line option for GBM treatment to improve the overall survival rate of patients [4, 5], however glioma cells eventually become metastatic and develop chemoresistance, which is an obvious block to the TMZ-therapeutic efficacy to GBM [6]. Approximately 55% of GBM patients are resistant to TMZ due to the methyl guanine methyl transferase DNA repair system [7] and acquired chemoresistance is a severe limitation to this therapy with more than 90% of recurrent gliomas showing no response to a second cycle of chemotherapy [8]. Although growing numbers of studies have focused on exploring the molecular modulatory network involved in investigating the effective therapeutic targets to overcome chemoresistance, it is still vital to elucidate the molecular mechanisms underlying and suppressing resistance to TMZ to provide and develop potential novel molecular targets for GBM therapy.

Long non-coding RNAs (lncRNAs) are a diverse set of noncoding RNA transcripts with the length of more than 200 nucleotides involved in a major part of critical biological processes and influence on regulation of various cancer progressions including chemoresistance [9, 10]. Obviously, several studies have demonstrated that lncRNAs also play a functional role in the dysregulation of resistance to TMZ in glioma through different mechanisms [11, 12]. The lncRNA colorectal neoplasia differentially expressed (CRNDE) is transcribed from chromosome 16, sharing a bi-directional promoter with the adjacent IRX5 gene [13]. As research continues, an increasing interest in the function of CRNDE in glioma [14–16] reveals that CRNDE could modulate glioma cell growth and invasion with EGFR activation and through mTOR signaling, and promote malignant progression by attenuating miR-384/PIWIL4/STAT3 axis in GBM. These study tendencies suggest that CRNDE plays critical roles in tumorigenesis, cancer development and clinical prognosis in glioma. However, the reports on the role of CRNDE in chemotherapy resistance are rare. Thus far, the regulation of chemoresistance by CRNDE has only been verified in colorectal cancer [17] and liver cancer [18]. Therefore, the function and possible mechanism of CRNDE involved in the development of chemoresistance to TMZ in glioma, particularly in GBM, remain unclear and have not yet been elucidated.

Autophagy is an evolutionarily conserved catabolic process through which cellular material is delivered to lysosomes for degradation, leading to the maintenance of homeostasis [19]. Autophagy plays opposing, context-dependent roles in cancer, and interventions to both stimulate and inhibit autophagy have been proposed as therapies against cancer [20]. Recent studies have emphasized that autophagy has obviously played a crucial role in tumor biological behaviors including chemotherapy resistance, and inhibiting autophagy could significantly increase chemo-therapeutic efficacy [21]. Meanwhile, TMZ-induced autophagy is an important participator in the drug resistance of glioma [22, 23]. Recently, accumulating evidence has confirmed a paradoxical role of autophagy in chemotherapy against glioma. A previous study found that overexpression of miR-519a promotes autophagy and subsequently enhances the chemosensitivity of glioblastoma cells to TMZ [24]. Similarly, phosphorylation of ULK1 inhibits autophagy in glioma and promotes the development of resistance to TMZ [25]. Several lines of research have confirmed the protective roles of autophagy in glioma cells during TMZ-based chemotherapy. Degradation of SMAD7 ubiquitination promotes autophagy to induce epithelial–mesenchymal transition and resistance to TMZ in glioblastoma [26]. Analogously, clinical data demonstrated that combination treatment with TMZ and an autophagy inhibitor could improve the prognosis of patients [27]. However, the underlying mechanism connecting autophagy and drug resistance is still not clear.

In the present study, the clinicopathological features of CRNDE were assessed and the role of CRNDE in glioma cell viability, colony formation, proliferation, apoptosis, autophagy and ABCG2 expression induced by TMZ were performed in vitro and in vivo. Moreover, the correlation between CRNDE and PI3K/Akt/mTOR pathway was explored to identify the mechanism in regulating TMZ-resistance in glioma, which might provide a new therapeutic target for GBM treatment.

## Methods

### Clinical specimens and follow-up

All of 58 cases of glioma tissues and corresponding 30 cases of normal brain tissues were obtained from Xiangya Hospital, Central South University (Changsha, China), under informed consent provided by all patients from May 2015 to February 2016 with the approval of the Ethic Committee of the Xiangya Hospital (approval no. 201907855). Primary tumor and normal brain tissues were frozen in liquid nitrogen and stored until total RNAs were extracted. Clinical data were simultaneously obtained from the medical records (Table 1). The relative factors of overall survival of patients were analyzed by univariate and multivariate analyses using the Cox proportional hazards model (Table 2). Glioma samples were diagnosed by two pathologists blinded to patient data. The tumor response status was evaluated according to the Response Evaluation Criteria in Solid Tumors version 1.0 criteria. The tumor response status to TMZ treatment was evaluated and the patients were assigned with complete/partial response (CR/PR) and stable/progressive disease (SD/PD) in tumor measurements confirmed through repeat studies performed ≥ 4 weeks after the initial fulfillment of the response criteria.

**Table 1:** Correlation of the CRNDE expression with clinical and pathological characteristics in tissue samples from glioma patients

Characteristics	CRNDE expression		Total (n = 58)	χ^2^	*P* value
	Low (n = 23)	High (n = 35)			
Age (years)					
< 55	12	18	30	0.003	0.584
≥ 55	11	17	28		
Gender					
Male	13	21	34	0.069	0.502
Female	10	14	24		
Location					
Supratentorial	16	26	42	0.155	0.459
Subtentorial	7	9	16		
Tumor size (mm)					
< 50	15	13	28	4.381	0.034^*^
≥ 50	8	22	30		
Peritumoral edema range (mm)					
< 10	10	6	16	4.819	0.030^*^
≥ 10	13	29	42		
Histological grade					
Grade I–II	17	5	22	20.960	< 0.001^*^
Grade III–IV	6	30	36		
Postoperative recurrence					
Yes	8	23	31	5.337	0.020^*^
No	15	12	27		

^*^P < 0.05 was considered to be statically significant

**Table 2:** Univariate and multivariate Cox proportional hazards regression model analysis of factors related to overall survival for patients

Characteristics		Univariate analysis			Multivariate analysis		
		HR	95% CI	*P* value	HR	95% CI	*P* value
Age (years)							
< 55 vs. ≥ 55		0.947	0.628–1.298	0.683	–	–	–
Gender							
Male vs. Female		0.752	0.451–1.364	0.464	–	–	–
Location							
Supratentorial vs. Subtentorial		1.134	0.967–1.602	0.301	–	–	–
Tumor size (mm)							
< 50 vs. ≥ 50		0.828	0.754–8.976	0.832	–	–	–
Peritumoral edema range (mm)							
< 10 vs. ≥ 10		1.876	0.667–3.546	0.795	–	–	–
Histological grade							
I + II vs. III + IV		0.392	0.265–0.769	0.024^*^	0.492	0.328–0.893	0.040^*^
Postoperative recurrence							
No vs. Yes		0.487	0.291–0.896	0.039^*^	0.537	0.314–0.915	0.043^*^
CRNDE expression							
High vs. Low		2.468	1.109–4.654	0.002^*^	1.897	1.132–2.387	0.006^*^

^*^P < 0.05 was considered to be statically significant

### Cell culture and transfection

The human glioma cell lines (U87 and U251) were obtained from the Cell Bank of Shanghai Institutes of Biochemistry and Cell Biology, Chinese Academy of Sciences, and normal human astrocyte cells (NHA) were purchased from BNBIO (Beijing, China). The cells were maintained in Dulbecco’s modified Eagle’s medium supplemented with 10% fetal bovine serum, 100 U/mL penicillin, and 100 μg/mL streptomycin at 37 °C with 5% volume/volume CO_2_.

Three small interference RNAs (siRNAs) targeting CRNDE (target sequence shown in Additional file 1: Table S1), as well as a negative control (si-NC), were designed and synthesized (RiboBio, Guangzhou, China) for the in vitro knockdown experiments. In addition, sh-CRNDE plasmids and a respective non-targeting sequence (negative control, sh-NC) were synthesized (Life Technologies, Waltham, MA, USA) in vivo. The sequences of sh-CRNDE and sh-NC are shown in Additional file 1: Table S1. Lentivirus-mediated CRNDE-expressing vector (EX-V0543-Lv324) and negative control group vector-NC (EX-NEG-Lv324) were purchased from GeneCopoeia (Rockville, MD, USA).

For cell transfection, glioma cells were seeded in a six-well plate and pre-incubated in the culture medium without antibiotics overnight, until they reached 50% confluence. Cells were transfected with plasmids or siRNAs using Lipofectamine 2000 (Invitrogen, Carlsbad, CA, USA) according to the instructions provided by the manufacturer. Subsequently, stable cell lines were established and selected.

### Culture of primary glioblastoma cells

All the primary glioma samples collected had the informed consent of the patients. Primary glioblastoma samples (selected from the GBM tissues) were minced using a GentleMACS Dissociator (Miltenyi Biotec, Gladbach, Germany) and digested in 0.25% trypsin at 37 °C for 30 min. The digestion was terminated by adding a trypsin inhibitor, and the cells were passed through a 40 μm nylon cell strainer (352340; CORNING, NY, USA) to obtain single-cell suspensions. Next, the cells were cultured in Dulbecco’s modified Eagle’s medium/F12 containing 10% fetal bovine serum. The primary glioblastoma cells were tested through glial fibrillary acidic protein staining and subcutaneous implantation in nude mice.

### RNA isolation and quantitative reverse transcription-polymerase chain reaction (qRT-PCR)

Total RNA from the cells was harvested using the TriZol reagent (Invitrogen). The SYBR Green RT-PCR Kit (QIAGEN, Germantown, MD, USA) was used to perform the reverse transcription and qRT-PCR reactions. Parameters of thermal cycle were: 95 ℃ for 10 s, 45 cycles of 95 ℃ for 5 s, 60 ℃ for 10 s and 72 ℃ for 10 s, followed by extension at 72 ℃ for 5 min. Each reaction was repeated in triplicates followed manufacturer protocol. The relative mRNA expression levels were calculated using the 2^−△△Ct^ method and normalized to the housekeeping gene glyceraldehyde-3-phosphate dehydrogenase (GAPDH) (control). The sequences of the CRNDE and GAPDH primers are shown in Additional file 1: Table S1.

### Cell Counting Kit-8 (CCK-8) cell viability assay

Cell viability was determined using the CCK-8 (Dojindo Molecular Technologies, Kumamoto, Japan) assay according to the instructions provided by the manufacturer. Briefly, 3000 cells in 150 µL of medium per well were seeded into a 96-well plate and cultured for 1–3 days. Subsequently, 15 µL of CCK-8 was added to each well, and cells were further incubated for 3 h. Absorbance was subsequently measured at 450 nm using a microplate reader (Thermo Fisher Scientific, Waltham, MA, USA).

### Cellular colony formation assays

For colony formation assay, the transfected cells were placed in six-well plates in a triplicate manner in a concentration of 1000 cells per well. A total of 2 weeks later, cells were fixed in 4% paraformaldehyde for 15 min at room temperature and stained with 0.01% crystal violet dye at room temperature for 15 min. The colonies were counted by microscopy after 3 weeks.

### 5-ethynyl-2´-deoxyuridine (EdU) cell proliferation assay

Cell proliferation was measured using the EdU incorporation assay according to the instructions provided by the manufacturer. Briefly, 2000 cells in 150 µL of medium per well were seeded into a 96-well plate, and cultured for 4 h. Subsequently, the medium was replaced by 10 μM EdU DNA Cell Proliferation Kit (RiboBio, Guangzhou, China) and the cells were cultured for an additional 12 h. Thereafter, the cells were fixed with 4% formaldehyde for 30 min. Next, the cells were exposed to 100 μL of Apollo stain per well for 30 min and incubated with 5 μg/mL of 4',6-diamidino-2-phenylindole for 15 min to stain the cell nucleus. Finally, the cells were counted using a fluorescent microscope (Olympus, Tokyo, Japan).

### Flow cytometric analysis of apoptosis

Apoptosis was assessed by annexin-V and propidium iodide (PI) staining as previously described [2]. The Annexin V-FITC/PI apoptosis detection kit (BD PharMingen, USA) was used to label the harvested cells according to the instructions provided by the manufacturer. The FACSCalibur (BD Biosciences, Franklin Lakes, NJ, USA) flow cytometer was used to quantify the apoptotic and necrotic cells. Cells in the early apoptotic (Annexin V-positive and PI-negative) or late apoptotic (Annexin V-positive and PI-positive) phases were included in cell death determinations.

### Western blot analysis

Total proteins were extracted from the cells using radioimmunoprecipitation assay buffer with protease inhibitors (Beyotime Institute of Biotechnology, Guangzhou, China) and the concentrations were estimated. Subsequently, equal amounts of protein samples were subjected to 10% sodium dodecyl sulfate–polyacrylamide gel electrophoresis and transferred to polyvinylidene difluoride membranes. After blocking with bovine serum albumin, the membranes were incubated with primary antibodies at 4 °C overnight and the appropriate horseradish peroxidase-conjugated secondary antibody at room temperature for 2 h. Finally, the proteins were visualized using a chemiluminescence detection kit (Aidlab Biotechnology, Beijing, China) and quantified through the Image Lab 4.0 software (Bio-Rad Laboratories, Richmond, CA, USA). The relative integrated density values were calculated based on GAPDH as an internal control. The primary antibodies included caspase-3/cleaved caspase-3, PARP /cleaved PARP, Phospho-PI3K/PI3K, Phospho-AKT/AKT, Phospho-mTOR/mTOR, LC3I/II, Beclin 1, Atg5, p62, ABCG2, and GAPDH (Cell Signaling Technology, Danvers, MA, USA).

### Mouse tumor xenograft model and immunohistochemistry (IHC)

This experiment was performed as previously described [2]. The nude mice were purchased from Laboratory Animal Center of Xiangya School of Medicine, Central South University. The animals were maintained and identified in a specific pathogen-free facility. The cells were suspended at a concentration of 1 × 10^7^/mL in PBS, respectively. Subsequently, 200 μL of cancer cell suspension was subcutaneously injected into the dorsal flanks of male nude mice aged 4–6 weeks (six mice per group). Once tumors were palpable (approximately 50 mm^3^), these mice were treated with 5 μg/g TMZ in 25% DMSO saline solution through intraperitoneal injection (5 days per week × 4 weeks). After 28 days from the initial treatment with TMZ, the mice were sacrificed and necropsies were performed. Tumor growth was monitored by caliper measurement every 4 days for a total of 28 days. Tumor volume was calculated as follows: V = L × l^2^/2, where L and l represent the larger and the smaller tumor diameters, respectively. The animal experiments were conducted in accordance with the National Institutes of Health Guide for the Care and Use of Laboratory Animals, and approved by the Animal Experimental Committee of Xiangya School of Medicine, Central South University. Tumor tissues from animals were paraffin embedded and stained with ABCG2 (Cell Signaling Technology) according to standard immunohistochemistry protocols, as previously described [2]. The image analysis was performed and the total gray value was estimated using the GSM-2000P pathology image analysis system (Heima, Guangzhou, China).

### Statistical analysis

All results were presented as mean ± standard deviation (SD) from 3 independent experiments. The Student’s *t*-test and/or *χ*^*2*^ tests were performed for the comparison of data between groups. Overall survival curves were plotted according to the Kaplan–Meier method, and the log-rank test was used for comparison. Survival was counted from the day of the surgery. All of the differences were statistically significant at the P < 0.05 level. All statistical analyses were performed using the SPSS version 21.0 software (IBM Corp., Armonk, NY, USA) and GraphPad Prism software 5.0 (GraphPad Software, Inc., San Diego, CA, USA).

## Results

### LncRNA CRNDE expression is upregulated in glioma and correlates with poor prognosis and TMZ resistance

**Fig. 1:**
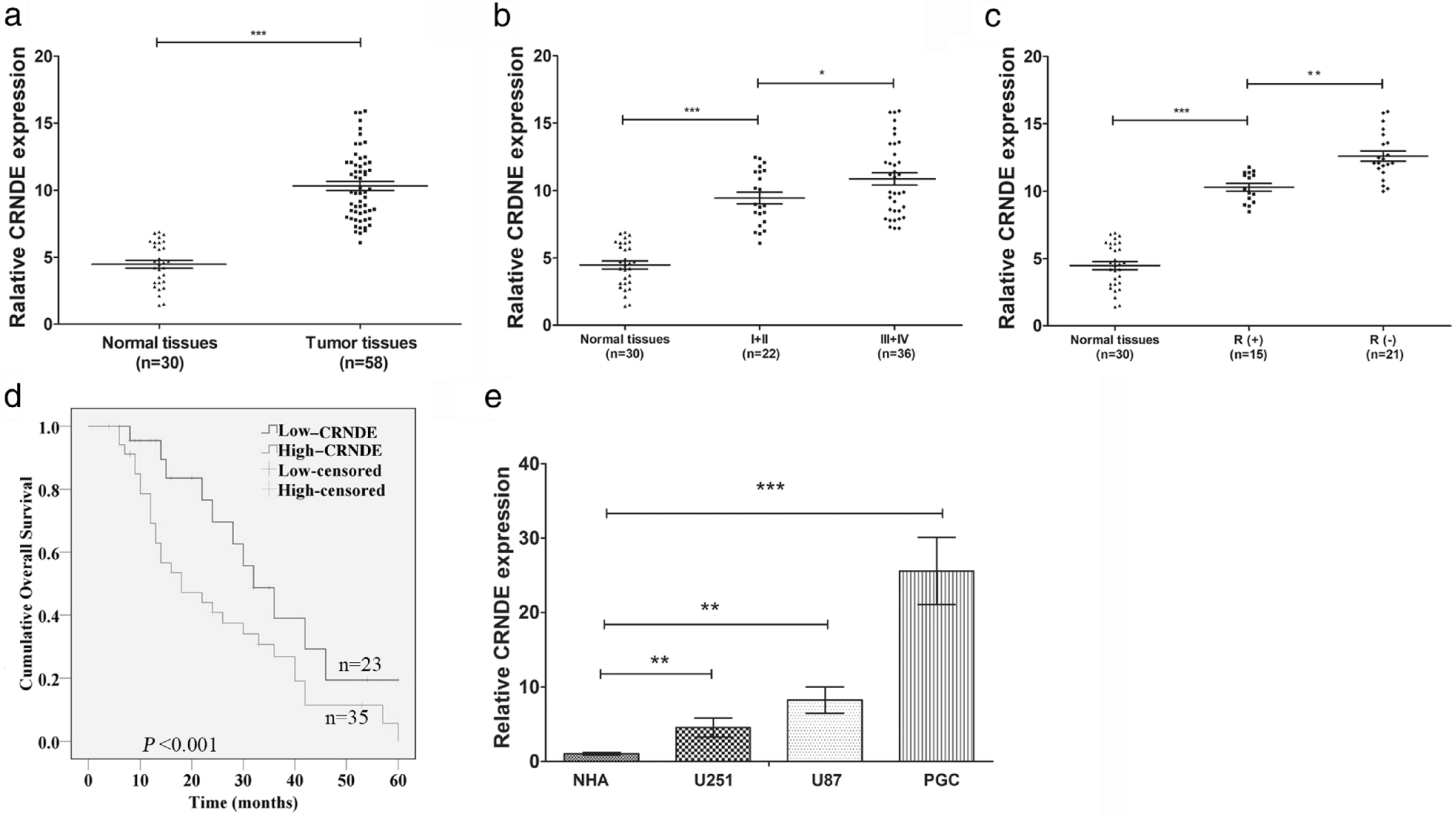
CRNDE expression in tissues and cells of glioma and the clinicopathological characteristics and prognosis of patients with glioma. **a** CRNDE expression in normal brain tissues and glioma tissues was determined by qRT-PCR. ^***^P < 0.001. **b** CRNDE expression was measured in normal brain tissues and glioma tissues at different pathological stage by qRT-PCR. ^*^P < 0.05, ^***^P < 0.001. **c** CRNDE expression was detected in glioma tissues showing response (R +) or no response (R-) to TMZ in high-grade tumors versus normal tissues. ^**^P < 0.01, ^***^P < 0.001. **d** Kaplan–Meier plot of overall survival of patients was stratified by CRNDE expression. **e** CRNDE expression was determined by qRT-PCR in three glioma cell lines (U251, U87 and PGC) versus a normal cell line (HNA). ^**^P < 0.01, ^***^P < 0.001. Data are presented as mean ± SD from three independent experiments

To analyze the expression of lncRNA CRNDE in glioma, the levels of CRDNE mRNA in 58 glioma specimens and 30 normal brain tissues were investigated by carrying out qRT-PCR. The expression of CRNDE was found to be significantly up-regulated in glioma tissues (Fig. 1a, P  < 0.001), and the expression level was positively correlated with the pathological grading of WHO stage (Fig. 1b, P < 0.05). Meanwhile, CRNDE expression was relatively higher in 21 patients with negative response (SD/PD) to TMZ compared with 15 cases with positive response (CR/PR) among all 36 cases of high-grade glioma (Grade III-IV) (Fig. 1c, P < 0.05). Further, the correlation of CRNDE expression with the clinicopathological characteristics of glioma patients was analyzed. The expression of CRNDE was measured and recorded to calculate the average expression of CRNDE in glioma patients. According to the average expression level of CRNDE, the patients were classified into low expression group (n = 23) and high expression group (n = 35) (Table 1). The clinicopathologic analysis revealed that CRNDE expression significantly correlated with tumor size (P = 0.034), peritumoral edema range (P = 0.030), histological grade (P < 0.001) and postoperative recurrence (P = 0.020) (Table 1). Patients with high CRNDE expression more correlated with larger size (≥ 50 mm), worse edema (≥ 10 mm), higher histological grade (Grade III–IV) and easier postoperative recurrence than those with low CRNDE expression group. Additionally, the factors related to overall survival and pathological characteristics for patients were analyzed using univariate and multivariate Cox proportional hazards regression model analysis. And the survival analysis demonstrated that histological grade (95% CI 0.328–0.893, P = 0.040), postoperative recurrence (95% CI 0.314–0.915, P = 0.043) and CRNDE expression (95% CI 1.132–2.387, P = 0.006) affecting survival time were statistically significant (Table 2). Moreover, the prognosis of these 58 glioma patients was followed up for 60 months. The median survival of patients with high CRNDE expression group was 19.2 months while that with low CRNDE expression group was 32.5 months (t = 2.412, P = 0.012). Kaplan–Meier survival analysis revealed that glioma patients with higher CRNDE level exhibited shorter overall survival time and worse prognosis than patients with lower expression of CRNDE (Fig. 1d, P < 0.001). Furthermore, the primary GBM cells (referred to as PGC) derived from GBM specimens with pathological diagnosis were isolated and cultured which were much closer to the truth of biological function and clinical effect of CRNDE in GBM. These PGCs were identified through immunofluorescence staining with glial fibrillary acidic protein antibody, an established molecular marker of glioma (Additional file 2: Figure S1). Subsequently, the expression level of CRNDE was detected in U251, U87 and PGC lines respectively by qTR-PCR. Compared with the normal gliocyte (NHA) cells, the levels of CRNDE were significantly upregulated in all glioma cell lines, especially in PGC cells (Fig. 1e,  P < 0.05). These data suggested that lncRNA CRNDE were upregulated in glioma tissues and cell lines, associated with the malignant phenotypes of glioma, correlated to poor prognosis of patients and TMZ resistance in glioma.

### Knockdown of CRNDE enhances chemosensitivity to TMZ, inhibits cell viability, decreases cell proliferation, facilitates cell apoptosis, and reduces the expression of ABCG2 in glioma cells

**Fig. 2:**
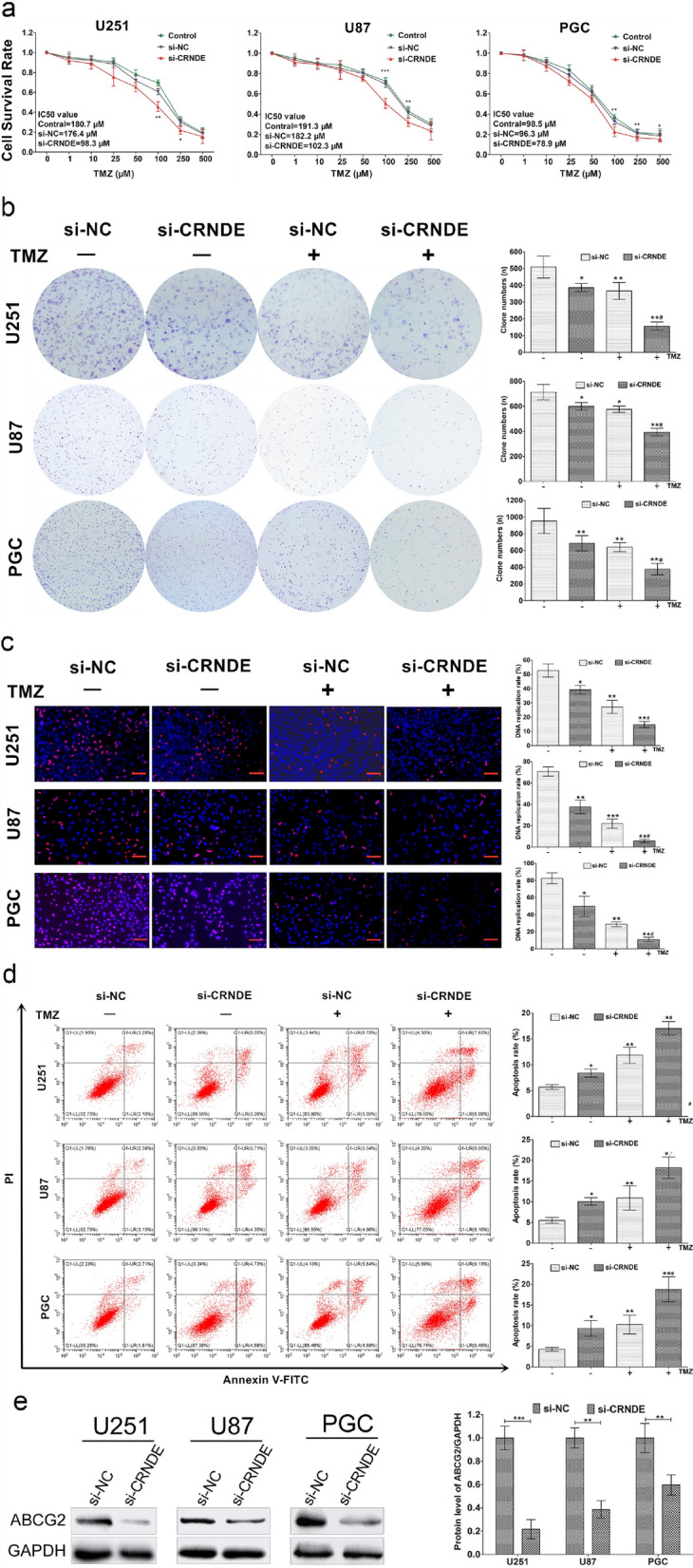
Knockdown CRNDE enhanced chemosensitivity to TMZ and reduced ABCG2 expression in vitro. **a** CCK-8 assays revealed cell viability with the exposure of TMZ depending on different doses for 72 h and IC_50_ values after si-NC or si-CRNDE transfection in three cell lines, respectively. ^*^P < 0.05, ^**^P < 0.01 and ^***^P < 0.001 represent si-CRNDE groups compared with si-NC groups, respectively. Colony formation assays (**b**) and EdU assays (**c**) showed cell proliferation ability of si-NC or si-CRNDE cells with or without exposure of TMZ at 100 μM for 72 h in three cell lines, respectively. ^*^P < 0.05, ^**^P < 0.01 and ^***^P < 0.001 compared with si-NC cells without TMZ treatment, ^*#^P < 0.05, ^**#^P < 0.01 compared with si-NC group cells with TMZ treatment. **d** Cell apoptosis analysis revealed cell apoptosis ability of si-NC or si-CRNDE cells with or without exposure of TMZ at 100 μM for 72 h in three cell lines, respectively. ^*^P < 0.05, ^**^P < 0.01 compared with si-NC cells without TMZ treatment, ^*#^P < 0.05, ^**#^P < 0.01 compared with si-NC group cells with TMZ treatment. Scale bars = 100 μΜ. **e** Western blot detected the ABCG2 protein expression after si-NC or si-CRNDE transfection in three cell lines treated with TMZ at 100 μM for 72 h, respectively. Data represent mean ± SD from 3 independent experiments

To assess the biological role of CRNDE in chemosensitivity in glioma, the impact of altered CRNDE expression in glioma cell lines was evaluated. Due to the CRNDE expression was upregulated in glioma specimens and cell lines, the silencing of CRNDE was performed by transfecting si-CRNDE-1, si-CRNDE-2, and si-CRNDE-3 transcripts into three cell lines. Since only the si-CRNDE-2 could notably reduce the expression of CRNDE in all lines (Additional file 3: Figure S2), si-CRNDE-2 was used in the subsequent experiments to decrease the CRNDE expression. Following the transfection of si-CRNDE-2, all three cell lines were treated with a series of TMZ doses (i.e., 0, 1, 10, 25, 50, 100, 250, and 500 μM) for 72 h. Subsequently, the cell viability was monitored by using the CCK-8 assay. The results indicated that compared with that reported in the empty control group (si-NC), knockdown of CRNDE dramatically restrained cell viability with relatively lower IC_50_ values exposed to TMZ in a dose-dependent manner for 72 h in all cell lines respectively (Fig. 2a, P  < 0.05). Meanwhile, based on the IC_50_ value of si-CRNDE group in three cell lines (98.3 μM, 102.3 μM and 78.9 μM, respectively, Fig. 2a) and referred to similar studies [28, 29], we chose the TMZ concentration of 100 μM for subsequent experiments. Then, the effect of CRNDE expression on cell proliferation was assessed by clone formation assays in three cell lines treated with or without the treatment of TMZ at 100 μM for 72 h. The results revealed that the number of colonies was reduced in silence CRNDE group compared with control group without TMZ treatment (Fig. 2b, P < 0.05). Furthermore, the number of colonies was much more decreased in si-CRNDE groups than si-NC groups after TMZ exposure in three cell lines (Fig. 2b, P < 0.05). Simultaneously, EdU assay was applied to further assess the influence of CRNDE expression on cell proliferation. The percentage of EdU-positive cells was significantly decreased in si-CRNDE groups when compared with that of si-NC groups, which was more evident after TMZ treatment (Fig. 2c, P < 0.05) implying that the silence of CRNDE expression could inhibit cell proliferation after treatment with TMZ. Additionally, to further evaluate the influence of CRNDE expression on cell survival, cell apoptosis analysis was implemented in glioma cells with the same treatment. The results showed that both of the rate of apoptosis was significantly increased in the si-CRNDE groups versus the si-NC groups without or with TMZ treatment, which was more significant with TMZ treatment (Fig. 2d, P < 0.05) revealing knockdown of CRNDE could diminish the glioma cell survival. Besides, considering the multidrug resistance (MDR) that occurs in cancer cells is a major obstacle to efficient chemotherapy for tumors [30], the expression of certain ATP-binding cassette (ABC) transporters which are mainly influenced the MDR in chemotherapy included ABCB1, ABCC1 and ABCG2 were assessed in theses cell lines by qRT-PCR with the TMZ at 100 μM for 72 h. Only the expression of ABCG2 mRNA was found to be significantly decreased in CRNDE silenced groups in all three cell lines (Additional file 4: Figure S3). Western blot analysis was further examined to explore whether the drug-resistant protein ABCG2 accounts for CRNDE-mediated resistance to TMZ. The results showed that silencing of CRDNE significantly downregulated expression of ABCG2 compared with the control group in protein level with the TMZ treatment in all three cell lines (Fig. 2e, P < 0.01). Taken together, these data demonstrated that knockdown of lncRNA CRNDE could enhance chemosensitivity to TMZ, inhibit cell viability, decrease cell proliferation, facilitate cell apoptosis, and reduce the expression of ABCG2 in glioma cells.

### Knockdown of CRNDE expression suppresses TMZ-induced autophagy in glioma cells

**Fig. 3:**
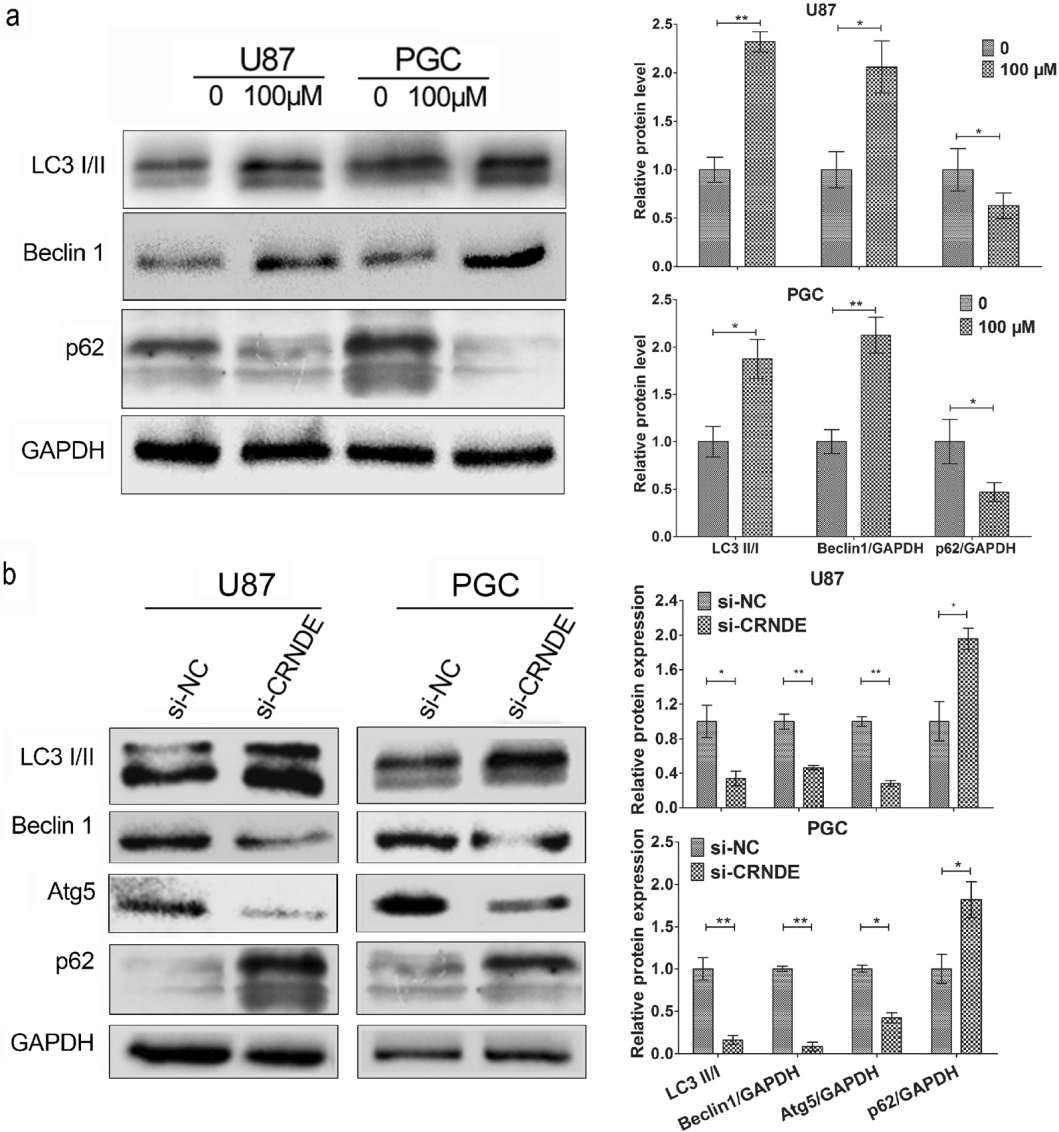
Knockdown CRNDE suppressed the TMZ-induced autophagy in glioma cells. **a** Western blot revealed the rate of protein LC3 II/I, protein levels of Beclin 1 and p62 in autophagic flux after treated with TMZ at 100 μM for 72 h in U87 and PGC lines. ^*^P < 0.05, ^**^P < 0.01. **b** Western blot showed the rate of protein LC3 II/I, protein levels of Beclin 1, Atg5 and p62 after CRNDE knockdown with the same treatment in U87 and PGC lines. ^*^P < 0.05, ^**^P < 0.01. Data are presented as mean ± SD from three independent experiments

Previous studies pointed out that TMZ could induce autophagy and TMZ-induced autophagy inhibition could improve the efficacy of TMZ therapy in GBM [22, 23], which implied that autophagy plays a potential role in chemoresistance of glioma. Therefore, the effect of CRNDE on the autophagy-related pathway in glioma cells treated with TMZ was investigated. The effect of TMZ treatment in inducing autophagy in U87 and PGC cells were initially evaluated. The common and established markers of autophagic flux such as LC3, Beclin1 and p62 were detected by Western blot analysis after the treatment of TMZ at 100 μM for 72 h. The activation of autophagy was evidenced by the increased levels of LC3 II/I and Beclin 1 proteins and decreased levels of p62 protein (Fig. 3a, P < 0.05). Furthermore, the expression of CRNDE was knocked down through the transfection of si-CRNDE in both cells to evaluate the CRNDE expression on the influence of TMZ-induced autophagy. The autophagic flux markers were subsequently detected after exposure to 100 μM TMZ for 72 h by Western blot analysis. Notably, the results verified that the protein levels of LC3 II/I, Beclin 1, and Atg5 were suppressed, whereas those of p62 were increased in the si-CRNDE groups compared with the si-NC groups in both cell lines (Fig. 3b, P < 0.05). Collectively, these findings emphasized that TMZ could activate the autophagy and downregulation of CRNDE might suppress the TMZ-induced autophagy in glioma cells.

### CRNDE regulates TMZ-induced autophagy through PI3K/Akt/mTOR pathway in glioma cells

**Fig. 4:**
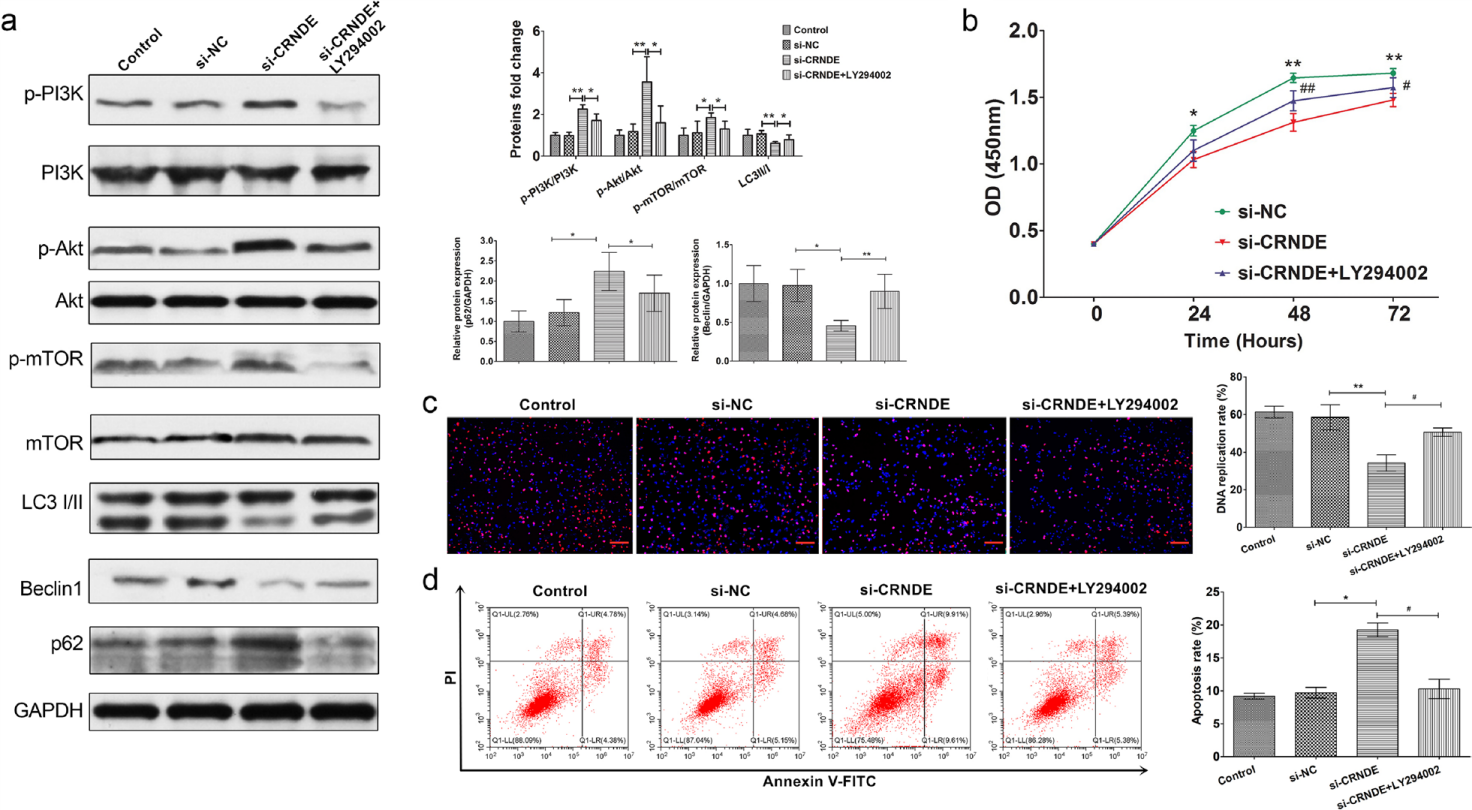
CRNDE regulated TMZ-induced autophagy through PI3K/Akt/mTOR pathway in glioma cells. **a** Western blot revealed the protein levels of p-PI3K/PI3K, p-Akt/Akt and p-mTOR/mTOR in PI3K/Akt/mTOR pathway and LC3 II/I, Beclin 1 and p62 in autophagy after CRNDE knockdown and addition with LY294002 with exposure of TMZ at 100 μM for 72 h in PGC line. ^*^P < 0.05, ^**^P < 0.01. **b** CCK-8 assays the cell viability after CRNDE knockdown and addition with LY294002 with exposure of TMZ at 100 μM for different hours in PGC line. ^*^P < 0.05, ^**^P < 0.01 represent si-CRNDE groups were compared with si-NC groups, respectively. ^#^P < 0.05, ^##^P < 0.01 represent si-CRNDE + LY294002 groups were compared with si-CRNDE groups, respectively. **c** EdU assays detected the cell proliferation after CRNDE knockdown and addition with LY294002 with exposure of TMZ at 100 μM for 72 h in PGC line. ^**^P < 0.01 represent si-CRNDE group was compared with si-NC group. ^#^P < 0.05 represent si-CRNDE + LY294002 groups was compared with si-CRNDE group. Scale bars = 100 μΜ. **d** Cell apoptosis analysis revealed cell apoptosis ability after CRNDE knockdown and addition with LY294002 with exposure of TMZ at 100 μM for 72 h in PGC line. ^*^P < 0.05 represent si-CRNDE group was compared with si-NC group. ^#^P < 0.05 represent si-CRNDE + LY294002 groups was compared with si-CRNDE group. Data represent mean ± SD from 3 independent experiments

The PI3K/Akt signaling pathway participates in the regulation of autophagy in anticancer therapy [31]. To elucidate the potential mechanism underlying autophagy exerting its effects on glioma cells, whether CRDNE had an impact on the PI3K/Akt signaling pathway and consequently modulated TMZ-induced autophagy were explored. After the same treatment in PGC cells, the expression of key markers in the PI3K/Akt pathway and autophagic flux at the protein level were monitored by Western blot analysis. Obviously, the depletion of CRNDE significantly upregulated the level of phosphorylate of PI3K, Akt, and mTOR, respectively to activate the signaling pathway. Simultaneously, it reduced the levels of LC3 II/I and Beclin 1 and increased that of p62 to suppress the autophagic flux in PGC cells compared with the control group and si-NC control group (Fig. 4a, P < 0.05). Additionally, to further confirm these findings, LY294002, a synthetic compound that was designed as a specific inhibitor for PI3K [32], was applied in si-CRNDE cells (si-CRNDE + LY294002 group). The data verified that the levels of phosphorylate of PI3K, Akt, and mTOR were reduced along with an increase in the levels of LC3 II/I and Beclin 1 and a decrease in that of p62 in this group compared with the si-CRNDE group (Fig. 4a, P < 0.05). These results demonstrated that LY294002 could synchronously restore the upregulation of the PI3K/Akt/mTOR pathway and inhibition of TMZ-induced autophagy. Moreover, the cell viability was assessed by CCK-8 assay in PGC cells with the treatment of TMZ at 100 μM for different hours. The results revealed that the cell viability was increased in si-CRNDE + LY294002 group when compared with the si-CRNDE group, suggesting that LY294002 could reverse the cell viability suppressed by the knockdown of CRNDE expression (Fig. 4b, P < 0.05). Additionally, EdU assay were further confirmed that LY294002 could significantly reverse the decreased cell proliferation by knockdown of CRNDE in PGC cells with the treatment of TMZ at 100 μM for 72 h (Fig. 4c, P < 0.05). Similarly, the influence of LY294002 on cell apoptosis was examined with the same treatment in PGC cells. The flow cytometry showed the LY294002 significantly reversed the upregulated cell apoptosis by the silence of CRNDE expression (Fig. 4d, P < 0.05). Meanwhile, the cell apoptosis was confirmed by Western blot analysis emphasizing that the levels of cleaved caspase-3 and cleaved PARP were upregulated by the silencing of CRNDE expression and then suppressed by the reversion of LY294002 (Additional file 5: Figure S4, P < 0.05). Together, all of these above results demonstrated that the altered expression of CRNDE could in some way regulate the TMZ-induced autophagy through the activity of PI3K/Akt/mTOR signaling pathway in glioma cells.

### Knockdown CRNDE enhances sensitivity to TMZ and reduces ABCG2 expression in vivo

**Fig. 5:**
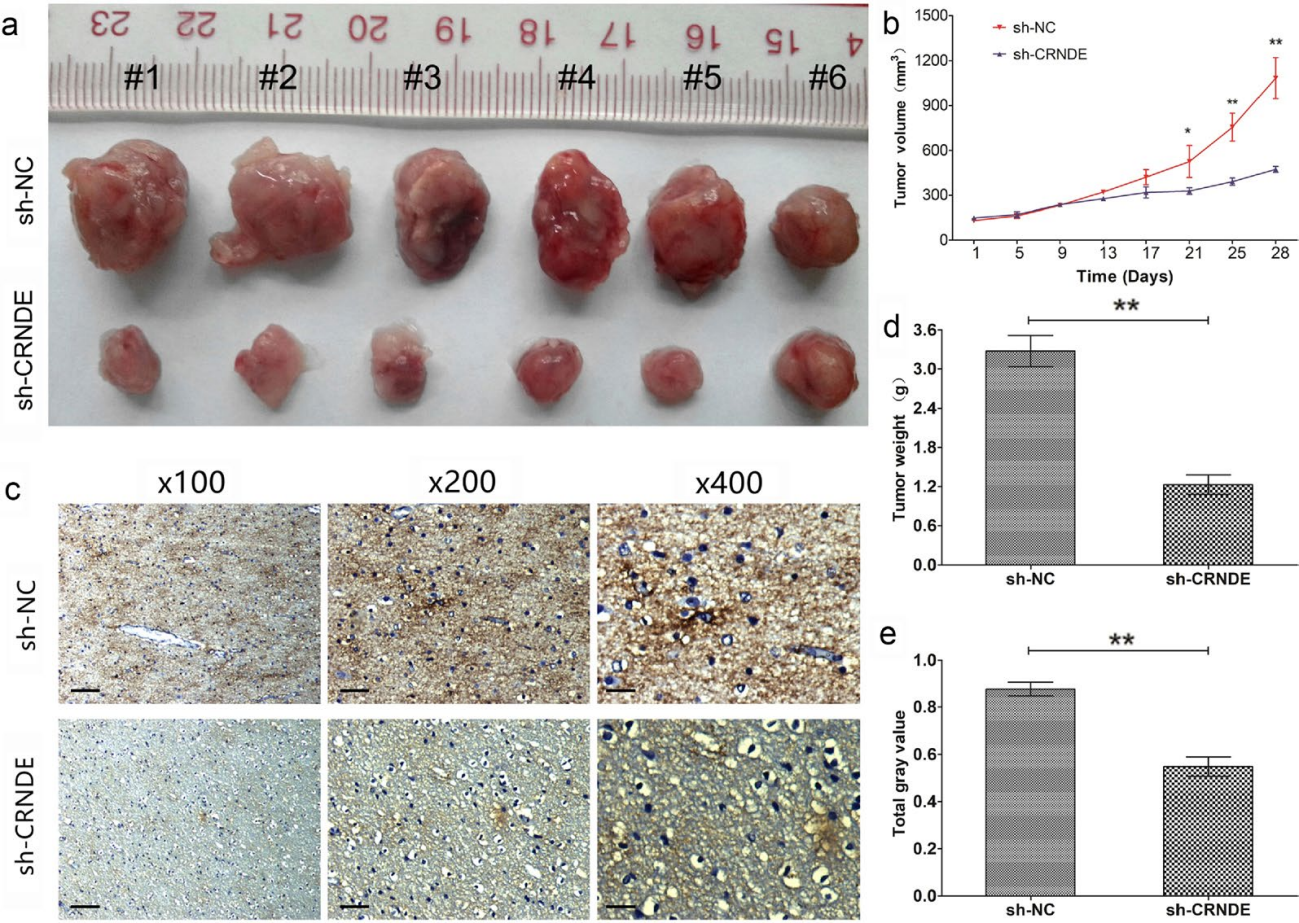
Knockdown CRNDE enhanced sensitivity to TMZ and reduced ABCG2 expression in vivo. **a** Representative images of xenograft tumors produced by CRNDE silenced or control cells in nude mice with TMZ treatment. **b** Tumor growth volume curve of the CRNDE knockdown subcutaneous PGC xenografts. ^*^P < 0.05, ^**^P < 0.01. **c** Tumor growth weights of the CRNDE knockdown subcutaneous PGC xenografts. ^**^P < 0.01. **d** Representative IHC images of ABCG2 expression in subcutaneous xenograft tumor tissue. Original magnification ×100 (Scale bars = 100 μΜ), X200 (Scale bars = 50 μΜ) and X400 (Scale bars = 25 μΜ). **e** The analysis of total gray value. ^**^P < 0.01. Data are presented as mean ± SD from three independent experiments. ^**^P < 0.01

To further determine the effect of altered CRNDE expression on the sensitivity of glioma cells to TMZ in vivo, PGC cells with knockdown expression of CRNDE by transfecting with sh-CRNDE or appropriate control cells by transfecting with sh-NC, subcutaneously injected into nude mice. After 4 weeks, the mice injected with PGC cells of sh-CRNDE showed a significant reduction in tumor volume (Fig. 5a, b, P < 0.05) and weight (Fig. 5c, P < 0.05) compared with the empty control mice injected with sh-NC cells after treatment with TMZ (5 μg/g). More deeply, IHC analysis of the tumors obtained from the mice demonstrated that the expression of ABCG2 was notably reduced in the sh-CRDNE group compared with the sh-NC group after treatment with TMZ (Fig. 5d, e, P < 0.05). Overall, these data in vivo further verified that the altered expression of CRNDE played a crucial biological role in chemosensitivity to TMZ in glioma and potentially related with the regulation of ABCG2.

## Discussion

Glioma is the most common and intractable type of intracranial tumors in adults and is associated with a poor prognosis due to the highly invasive growth pattern and frequent resistance to chemoradiotherapy [1]. As the most malignant histopathological type, GBM is almost invariably fatal with an overall survival of approximately only 1 year [2, 3]. TMZ-based chemotherapy, as the first-line option, is the routine and crucial therapy against GBM followed by regular surgical excision [4, 5]. Although the administration of TMZ improves the overall survival rate in patients with GBM in the clinical setting, the eventual occurrence of chemoresistance markedly impairs the efficacy of treatment against GBM [6]. Therefore, investigation of the underlying mechanism of chemoresistance is urgently warranted.

LncRNAs are a diverse set of noncoding RNA transcripts which play fundamental roles in the pathological processes related to tumorigenesis, invasion, metastasis, and chemoresistance in glioma [11, 12, 33, 34]. LncRNA CRNDE is dysregulated in several types of cancer and verified to be a promoter in glioma. Overexpression of CRNDE promotes the growth and invasion of glioma cells through mTOR signaling [16]. Meanwhile, the expression of CRNDE positively correlates with EGFR activation to modulate glioma cell growth [14]. Moreover, through attenuating the miR-384/PIWIL4/STAT3 axis, CRNDE promotes malignant progression in glioma cells [15]. Nevertheless, reports on the role of CRNDE in the development of resistance to chemotherapy in tumors are rare. While, the specific target and mechanism of CRNDE in chemoresistance to GBM have not yet been reported.

In this study, lncRNA CRNDE was confirmed the upregulation in human glioma tissues compared with normal brain tissues. Meanwhile, the expression of CRNDE was positively correlated with the histopathological stage of glioma. Moreover, in patients with high-grade glioma, the expression of CRNDE was significantly higher in glioma tissues not showing response to chemotherapy with TMZ than in those showing response. In addition, the expression of CRNDE in U251, U87, and PGC cells were investigated and found similar results, especially in the PGC. Furthermore, clinicopathological characteristics analysis verified that the glioma patients with high expression level of CRNDE potentially had a relatively high risk of larger tumor size, worse peritumoral edema range, higher histological grade and more frequent recurrence versus those with lower expression level. Importantly, the patients with higher expression of CRNDE were associated with worse outcomes and shorter survival time versus those who had lower expression. These findings demonstrated that the expression of CRNDE was an independent factor of poor prognosis for patients with glioma and potentially associated with the development of TMZ-based chemoresistance in glioma. Thereby, the CRNDE was surmised to participate in chemoresistance to influence the prognosis for patients with glioma.

Subsequently, to further validate these findings, the expression of CRNDE was altered and conducted CCK-8, colony formation, and EdU assays, as well as flow cytometry to detect the cell functions affected by CRNDE in response to treatment with TMZ. The results revealed that silencing of CRNDE enhanced the sensitivity to treatment by suppressing cell viability, clone formation, and proliferation, as well as promoting cell apoptosis in vitro, and reduced tumorigenesis in vivo without the exposure of TMZ, while these trends were more significant in response to TMZ treatment. Additionally, knockdown of CRNDE dramatically lowered IC_50_ values exposed to TMZ in a dose-dependent manner. Consistent with these results, CRNDE was considered to be deeply related to the development of resistance to TMZ in GBM cells. Moreover, the altered expression of CRNDE improved sensitivity to TMZ. Similar to our findings, in colorectal cancer, CRNDE knockdown would induce the repression of cell proliferation, reduction of chemoresistance, and inhibition of Wnt/β-catenin signaling by increasing the expression of miR-181a-5p [17]. Notably, downregulation of CRNDE could suppress drug resistance of liver cancer cells by increasing microRNA-33a expression and decreasing HMGA2 expression [18].

The development of multidrug resistance (MDR) is a major problem impeding the effectiveness of chemotherapy against cancer [30]. And one of the crucial mechanisms of chemoresistance in glioblastoma is the increased expression of certain adenosine triphosphate-binding cassette (ABC) transporters, including the drug resistance protein ABCG2 in MDR [30]. ABCG2 could determine the response of glioblastoma to TMZ [35], and its inhibition increases sensitivity to TMZ and suppresses the growth of TMZ-resistant glioma [36]. In our study, ABCG2 was verified to be positively regulated by the altered expression of CRNDE in functional experiments both in vitro and in vivo, which reminded us that the regulation of TZM-based chemoresistance by CRNDE may be associated with MDR, especially ABCG2. In recent literature, ABCG2 co-expressed with lncRNA CTD-2589M5.4 similarly changed in both of the multidrug resistant ovarian cancer cell lines and colon cancer cell lines [37]. However, the specific molecular mechanism involved in the regulation of MDR by CRNDE warrants further investigation in the future. At least, these results could imply that CRNDE was potentially related with the TMZ-resistant in GBM cells and the altered CRNDE expression might improve sensitivity to TMZ in glioma.

Autophagy promotes cell survival during nutrient depletion and is essential for maintaining cellular homeostasis by degrading damaged organelles and proteins [19]. Previous studies revealed that TMZ induces autophagy and inhibition of TMZ-induced autophagy improves the efficacy of TMZ against glioblastomas [22, 23], implying a potential role in the development of drug resistance of glioma. Although the evidence regarding the role of autophagy in TMZ-induced cytotoxicity is inconsistent [38], clinical data have substantiated that the combination treatment with TMZ and an autophagy inhibitor improved the prognosis of patients [23, 27]. Therefore, the autophagy, which could be evoked by exposure to TMZ with increased conversion from LC3 I to II, Beclin 1, and suppressed accumulation of p62, was firstly identified in our further analysis of the mechanism involved in this process. Meanwhile, autophagy-relevant in drug-resistance has been featured in chemotherapy recently in literature [26]. Consequently, the influence of CRNDE expression on the autophagic flux-related pathway in TMZ-treated glioma cells was determined. The results emphasized that silencing of CRNDE could impair the TMZ-induced autophagy in GBM cells through a reduction in the protein levels of LC3 II/I, Beclin 1, and Atg5, and an increase in those of p62. A recent study was reported that the lncRNA LINC00470 in GBM exosomes could inhibit autophagy and enhance the proliferation of glioma cells through binding to miR-580-3p to regulate WEE1 expression [39]. Similarly, silencing lncRNA DLEU1 could suppress TMZ-activated autophagy via regulating the expression of P62 and LC3, and promote sensitivity of glioma cells to TMZ by triggering apoptosis [40]. According to our findings in the current study, treatment with TMZ increased autophagy in GBM cells, and knockdown of CRNDE antagonized the activation of TMZ-induced autophagy in these cells.

Further, the potential molecular mechanism underlying the CRNDE-mediated inhibition of TMZ-induced autophagy was deciphered. The PI3K/Akt/mTOR signaling pathway was explicit to participate in the regulation of cell proliferation, migration, invasion, mitochondrial dysfunction, induction of autophagy in glioma [31, 41, 42] and anticancer therapy related to autophagy in cancers [43]. In our further study, the silencing of CRNDE contributed to the induced autophagy in glioma cells through upregulation of the PI3K/Akt/mTOR pathway, accompanied by the suppression of cell viability, proliferation, and apoptosis under treatment with TMZ. Moreover, the results indicated that the induced autophagy and cellular effects could be restored by treatment with the PI3K inhibitor LY294002. In the present study, we blocked PI3K by LY294002, resulting in the inactivation of the PI3K/Akt/mTOR pathway and reactivation of autophagy. These findings demonstrated that CRNDE may enhance the activation of TMZ-induced autophagy in a PI3K-dependent manner. More intriguingly, suppressing PI3K also reversed the increased chemosensitivity to TMZ induced by the knockdown of CRNDE in vitro. The literature reported that CRNDE promoted cell proliferation through PI3K/Akt signaling in hepatocellular carcinoma and non-small cell lung carcinoma [44, 45]. While, a recent study revealed that lncRNA FOXD1-AS1 could strengthen the interaction of eIF4E with eIF4G by activating the PI3K/AkT/mTOR pathway to promote gastric cancer progression and cisplatin resistance [46]. Homoplastically, lncRNA LINC00665 was confirmed to induce acquired resistance to gefitinib through recruiting EZH2 and activating PI3K/Akt pathway in non-small-cell lung cancer [47]. These findings showed that CRNDE regulates the activation of PI3K/Akt/mTOR signaling to reduce autophagy and enhance the sensitivity to TMZ in GBM cells.

## Conclusions

In conclusion, lncRNA CRNDE was associated with the malignant phenotypes of glioma and a poor prognosis for patients with GBM, which were firstly demonstrated in our integrated approach. The expression of CRNDE was upregulated in patients who were resistant to TMZ, and closely associated with the response status to treatment with TMZ. Meanwhile, the mechanism underlying the regulation of chemoresistance to TMZ by CRNDE in GBM in vitro and in vivo was identified in our study. Silencing of CRNDE enhanced the sensitivity to TMZ in GBM cells by reducing autophagy and highly correlated with the drug resistance protein ABCG2. The reduction of autophagy was attributed to the activation of the PI3K/Akt/mTOR signaling pathway; however, more detailed mechanisms of CRNDE in glioma needed to be revealed. A preclinical basis for the use of CRNDE as a reliable clinical predictor of outcome and prognosis in patients with GBM was provided in our findings. Moreover, CRNDE can be utilized as a potential biomarker for predicting response to treatment with TMZ and a new therapeutic target for enhancing sensitivity to TMZ in GBM.

## Supplementary Information

**Additional file 1: Table S1.** The sequences for the study.

**Additional file 2: Fig. S1.** The patient-derived glioma primary cells were identified by immunofluorescence staining with GFAP antibody. Scale bars = 20 μΜ.

**Additional file 3: Fig. S2.** The expression of CRNDE was measured after transfection with Si-CRNDEs or Si-NC by qRT-PCR in U251, U87 and PGC lines. ^*^P < 0.05, ^**^P < 0.01, ^***^P < 0.001. Data represent mean ± SD from 3 independent experiments.

**Additional file 4: Fig. S3.** The expression of ABC transporters, as ABCB1, ABCC1 and ABCG2 were detected by qRT-PCR after transfection with Si-CRNDE or Si-NC in three cell lines. ^*^P < 0.05, ^**^P < 0.01. Data represent mean ± SD from 3 independent experiments.

**Additional file 5: Fig. S4.** Western blot revealed the protein levels of cleaved caspase-3 and cleaved PARP in cell apoptosis after CRNDE knockdown and addition with LY294002 with exposure of TMZ at 100 μM for 72 h in PGC line. ^*^P < 0.05, ^**^P < 0.01. Data represent mean ± SD from 3 independent experiments.

## Data Availability

The datasets used or analyzed during the current study are available from the corresponding author on reasonable request.

## Abbreviations

GBM: Glioblastoma multiforme
TMZ: Temozolomide
lncRNA: Long non-coding RNA
CRNDE: Colorectal neoplasia differentially expressed
CR/PR: Complete/partial response
SD/PD: Stable/progressive disease
PGC: Primary GBM cells
ABC: ATP-binding cassette
MDR: Multidrug resistance
CCK-8: Cell Counting Kit-8
EdU: 5-Ethynyl-2´-deoxyuridine
IHC: Immunohistochemistry
