# Supplementary material for: Knockdown lncRNA CRNDE enhances temozolomide chemosensitivity by regulating autophagy in glioblastoma
Source: Cancer Cell Int. 2021 Aug 28;21:456. doi: 10.1186/s12935-021-02153-x (PMC8399846; doi:10.1186/s12935-021-02153-x)
Supplement: Supplementary file 1 — Additional file 1: Table S1. The sequences for the study. [file 12935_2021_2153_MOESM1_ESM.docx]

Table S1 The sequences for the study

| Target sequence for CRNDE | Forward | CCGUUGGUCUUUGAAAUUUTT |
| --- | --- | --- |
|  | Reverse | AAAUUUCAAAGACCAACGGTT |
| Sequence for sh-CRNDE | Forward | GATCCAAAAAAGGAAGGAGGAGATTCTGAAGATCTCTTGAATCTTCAGAATCTCCTCCTTCC |
|  | Reverse | CACCGGAAGGAGGAGATTCTGAAGATTCAAGAGATCTTCAGAATCTCCTCCTTCCTTTTTG |
| Sequence for sh-NC | Forward | GATCCAAAAAATTCTCCGAACGTGTCACGTAATCTCTTGACGTGACACGTTCGGAGAAC |
|  | Reverse | CACCGTTCTCCGAACGTGTCACGTCAAGAGATTACGTGACACGTTCGGAGAATTTTTTG |
| Primers sequence for CRNDE | Forward | CTGTACTGGCTATTGGAAGGAG |
|  | Reverse | AATTTCAAAGACCAACGGCTG |
| Primers sequence for GAPDH | Forward | GCGCCCAATACGACCAAATC |
|  | Reverse | AATGGGCAGCCGTTAGGAAA |
