# Supplementary figures and images for: Knockdown lncRNA CRNDE enhances temozolomide chemosensitivity by regulating autophagy in glioblastoma
Source: Cancer Cell Int. 2021 Aug 28;21:456. doi: 10.1186/s12935-021-02153-x (PMC8399846; doi:10.1186/s12935-021-02153-x)

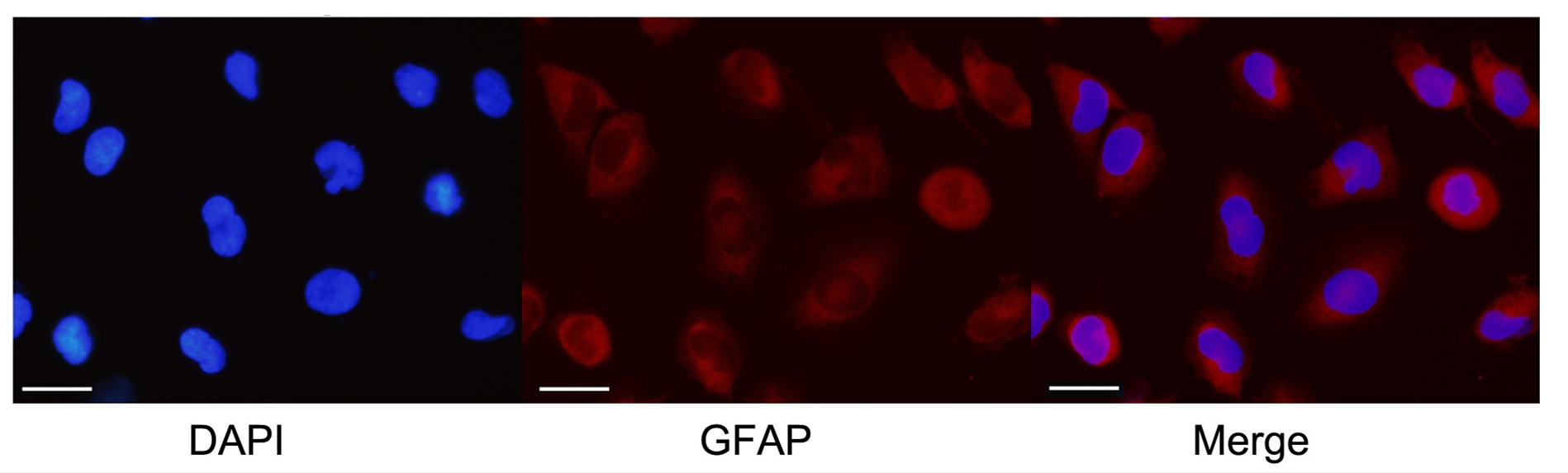

Supplement: Supplementary file 2 — Additional file 2: Fig. S1. The patient-derived glioma primary cells were identified by immunofluorescence staining with GFAP antibody. Scale bars = 20 μΜ. [file 12935_2021_2153_MOESM2_ESM.tif]

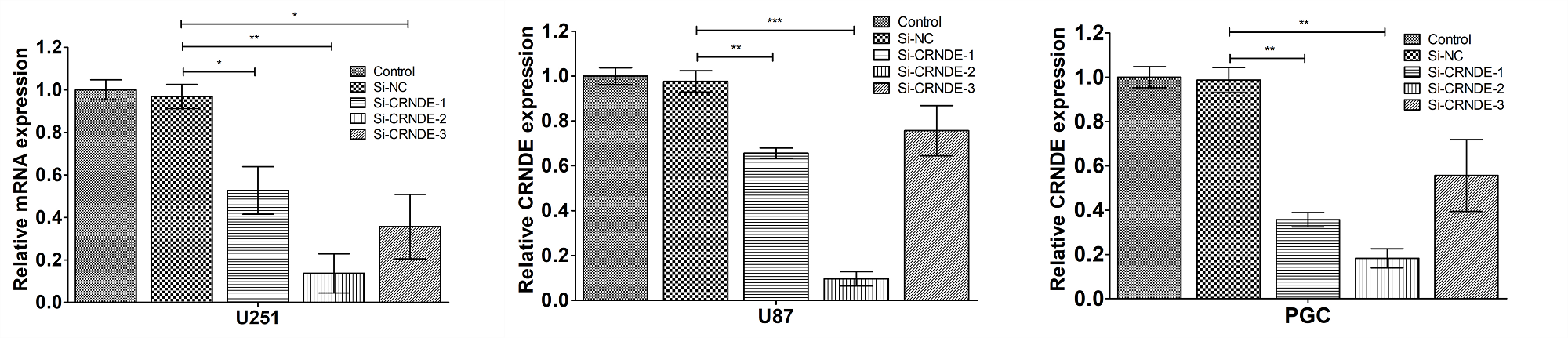

Supplement: Supplementary file 3 — Additional file 3: Fig. S2. The expression of CRNDE was measured after transfection with Si-CRNDEs or Si-NC by qRT-PCR in U251, U87 and PGC lines. *P < 0.05, **P < 0.01, ***P < 0.001. Data represent mean ± SD from 3 independent experiments. [file 12935_2021_2153_MOESM3_ESM.tif]

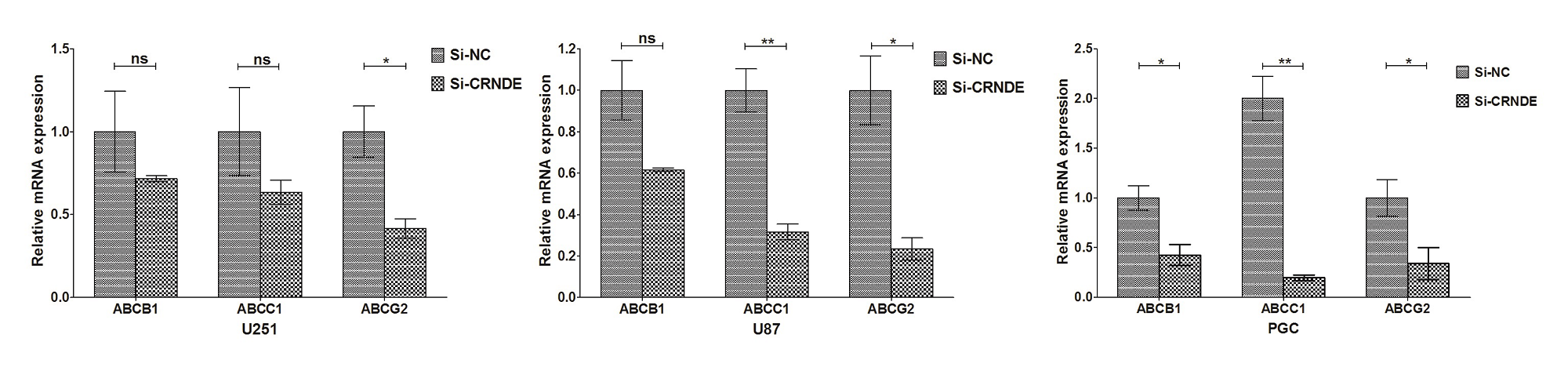

Supplement: Supplementary file 4 — Additional file 4: Fig. S3. The expression of ABC transporters, as ABCB1, ABCC1 and ABCG2 were detected by qRT-PCR after transfection with Si-CRNDE or Si-NC in three cell lines. *P < 0.05, **P < 0.01. Data represent mean ± SD from 3 independent experiments. [file 12935_2021_2153_MOESM4_ESM.tif]

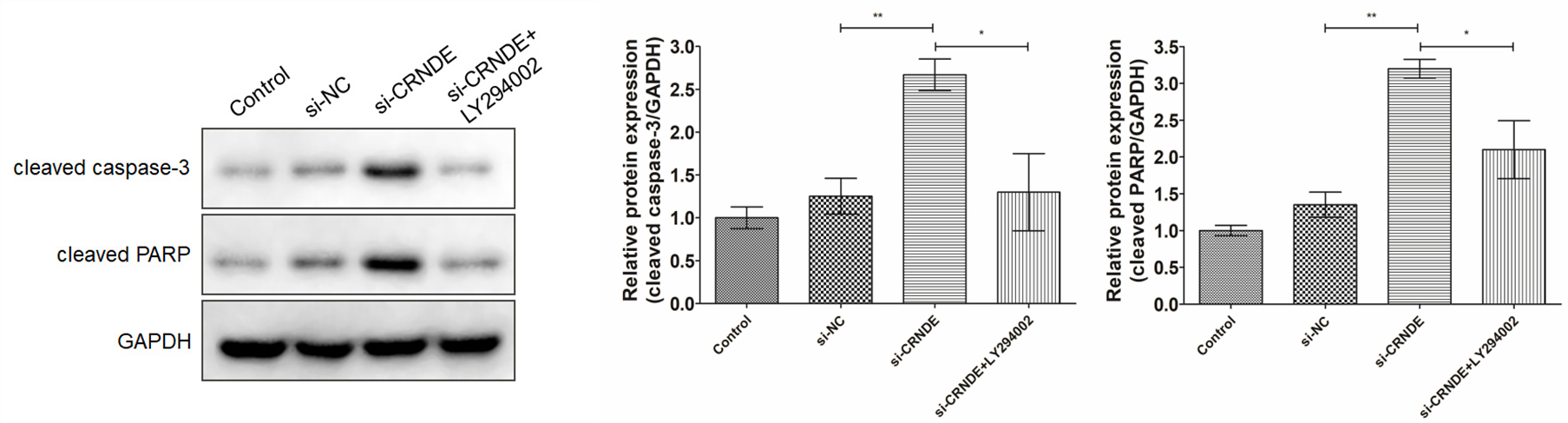

Supplement: Supplementary file 5 — Additional file 5: Fig. S4. Western blot revealed the protein levels of cleaved caspase-3 and cleaved PARP in cell apoptosis after CRNDE knockdown and addition with LY294002 with exposure of TMZ at 100 μM for 72 h in PGC line. *P < 0.05, **P < 0.01. Data represent mean ± SD from 3 independent experiments. [file 12935_2021_2153_MOESM5_ESM.tif]
